# Quantifying the visual-sensory landscape qualities that contribute to cultural ecosystem services using social media and LiDAR

**DOI:** 10.1016/j.ecoser.2018.03.022

**Published:** 2018

**Authors:** Derek B. Van Berkel, Payam Tabrizian, Monica A. Dorning, Lindsey Smart, Doug Newcomb, Megan Mehaffey, Anne Neale, Ross K. Meentemeyer

**Affiliations:** a ORISE Fellowship Participant, US EPA, Office of Research and Development, Research Triangle Park, Durham, NC, USA; b Center for Geospatial Analytics, North Carolina State University, Raleigh, NC 27695, USA; c College of Design, Campus Box 7701, North Carolina State University, Raleigh, NC 27695, USA; d Geosciences and Environmental Change Science Center, U.S. Geological Survey, Denver, USA; e Forestry and Environmental Resources, NC State University, USA; f U.S. Fish and Wildlife Service, Raleigh, NC, USA; g US EPA, Office of Research and Development, National Exposure Research Laboratory, Research Triangle Park, Durham, NC, USA

**Keywords:** Cultural ecosystem services, Spatial analysis, Coastal scenery, Social media

## Abstract

Landscapes are increasingly recognized for providing valuable cultural ecosystem services with numerous non-material benefits by serving as places of rest, relaxation, and inspiration that ultimately improve overall mental health and physical well-being. Maintaining and enhancing these valuable benefits through targeted management and conservation measures requires understanding the spatial and temporal determinants of perceived landscape values. Content contributed through mobile technologies and the web are emerging globally, providing a promising data source for localizing and assessing these landscape benefits. These georeferenced data offer rich *in situ* qualitative information through photos and comments that capture valued and special locations across large geographic areas. We present a novel method for mapping and modeling landscape values and perceptions that leverages viewshed analysis of georeferenced social media data. Using a high resolution LiDAR (Light Detection and Ranging) derived digital surface model, we are able to evaluate landscape characteristics associated with the visual-sensory qualities of outdoor recreationalists. Our results show the importance of historical monuments and attractions in addition to specific environmental features which are appreciated by the public. Evaluation of photo-image content highlights the opportunity of including temporally and spatially variable visual-sensory qualities in cultural ecosystem services (CES) evaluation like the sights, sounds and smells of wildlife and weather phenomena.

## 1. Introduction

Landscapes are increasingly recognized for their provision of valuable cultural ecosystem services (CES) as they support recreation and leisure activities linked to educational, inspirational, and spiritual values, and improved overall mental and physical health and well-being (Sandifer et al., 2015; van Zanten et al., 2016a). Highly valued landscapes often include cultural features (e.g., historical monuments) and potential activities (e.g., local foods, kayak rentals) that synergistically enhance and contribute to their uniqueness as special locations (Hausmann et al., 2016). Planning, environmental management, and tourism fields have long recognized the effects of values and perceptions on the way landscapes are used (Dorning et al., 2017). These perceptive lenses shape how different individuals use and appreciate the numerous non-material benefits of nature (e.g., for an overview of CES see Plieninger et al. (2013), Chan et al. (2012)). Significant advances have been made evaluating CES using respondents’ assessment of landscape photographs (Van and Verburg, 2014; van Zanten et al., 2016b), immersive virtual environments (Appleton and Lovett, 2003; Smith, 2015; Tabrizian et al., 2018), and participatory techniques (Brown and Fagerholm, 2015). Rarely do such surveys capture the diversity of appreciated landscapes as they are confined to specific locations and small samples of the population (van Zanten et al., 2016a; Sherrouse et al., 2011). Moreover, such studies may miss the multiple non-material factors that contribute to the way a landscape is valued and experienced, including iconic features, cultural and ecological elements (e.g., monuments or wildlife), and other sensory attributes (e.g., sounds or smells). Assessing landscape-scale variation in a wide range of values would improve our understanding of the diversity of specific locations that are appreciated for their CES.

The full range of CES provided by a landscape is notoriously difficult to evaluate at the landscape-scale due to the absence of detailed data regarding the cultural values people associate with a specific place and the diversity within the landscape mosaic (Plieninger et al., 2013; Chan et al., 2012). Data mined from social media show promise for evaluating these benefits by serving as empirical evidence of visited and appreciated landscapes at broad spatial scales. For example, social media have been leveraged to map and quantify CES (Sonter et al., 2016; Richards and Friess, 2015; Oteros-Rozas et al., 2017; Casalegno et al., 2013; Richards and Tunçer, 2018), characterize landscapes (van Zanten et al., 2016b; Tieskens et al., 2017), and estimate the number of visitors to protected areas (Wood et al., 2013; Heikinheimo et al., 2017).

While social media posts can provide information regarding cultural services, photos have the added benefit of documenting appreciated features in the photo content itself and sensory qualities that can be inferred through photo content (e.g., smell of the ocean, the song of a bird). Surprisingly, this photographic content has scarcely been investigated, with the exception of a few studies (Dunkel, 2015; Oteros-Rozas et al., 2017). For example, Oteros- Rozas et al. (2017) demonstrated that photographs volunteered via social media could be classified according to CES categories. Dunkel (2015) similarly found that volunteered photos offered unique insights into the photographers’ values, which could be inferred through the subject matter and composition of captured images (Dunkel, 2015). Assessing social media photographic content could add a new level of richness in our understanding of what constitutes a CES, by providing greater detail of appreciated features.

Photo content may also help in validating the location of photographic capture. Geographic misclassification can occur due to inaccuracies in recording the user’s position through mobile technologies (i.e., GPS signal loss) or errors in actual coordinate pinpointing by users on older website mapping-interfaces (Zielstra and Hochmair, 2013). To date, such errors have been assessed primarily in urban settings by measuring the distance between the volunteered image position and the manually corrected camera position of landmarks (Zielstra and Hochmair, 2013). Such a measure is unlikely to be feasible in landscape scale studies with large numbers of social media locations that lack distinctive landmarks. Alternatively, error evaluation can potentially be achieved by comparing the photographic content captured at these locations with verifiable spatial layers depicting similar attributes (e.g., land use) (Oteros-Rozas et al., 2017).

While photographs may capture targeted aspects of appreciation, it is increasingly acknowledged that the entire surrounding landscape context or the experienced environment contribute to CES delivery (van Zanten et al., 2016b). Calculating the viewshed is a potentially useful geographic tool that can capture how the environment is experienced. A viewshed is the 360° area that is visible from a discrete location (Vukomanovic et al., 2018). It includes all surrounding points within the line-of-sight of an assumed viewer’s location and excludes points that are obstructed by the terrain and other features (e.g., buildings, trees). Viewshed research has been instrumental in understanding scenic values associated with residential development (Vukomanovic and Orr, 2014) and the relationship between aesthetic values and landscape patterns (Schirpke et al., 2016). However, difficulty in identifying the subject of appreciation within the viewshed has unfortunately caused many studies to resort to a best guess regarding the precise location of appreciated areas (Schirpke et al., 2016; Yoshimura and Hiura, 2017). Combining georeferenced photos volunteered by social media users with viewshed analysis presents a unique opportunity to evaluate the landscape qualities and visible attributes associated with high valued areas (Yoshimura and Hiura, 2017).

In this study, our objective is to demonstrate a novel multi-method approach for capturing and understanding the visible-sensory landscape qualities that contribute to CES. We aim to evaluate the experienced environments of visitors to coastal North Carolina through the locations they volunteer via social media. In doing so, we will (1) test the ability of high-resolution LiDAR to define the visible area of these locations; (2) assess the applicability of social media photographs to enrich our understanding of CES; and (3) characterize the natural and built environments and activities that are highly photographed. We assume that locations with high numbers of photographs deliver numerous CES and hypothesize that our multi-method approach can better contextualize the recreational potential, aesthetic character, and uniqueness that likely characterize these areas.

## 2. Methods

### 2.1. Study area

**Fig. 1:**
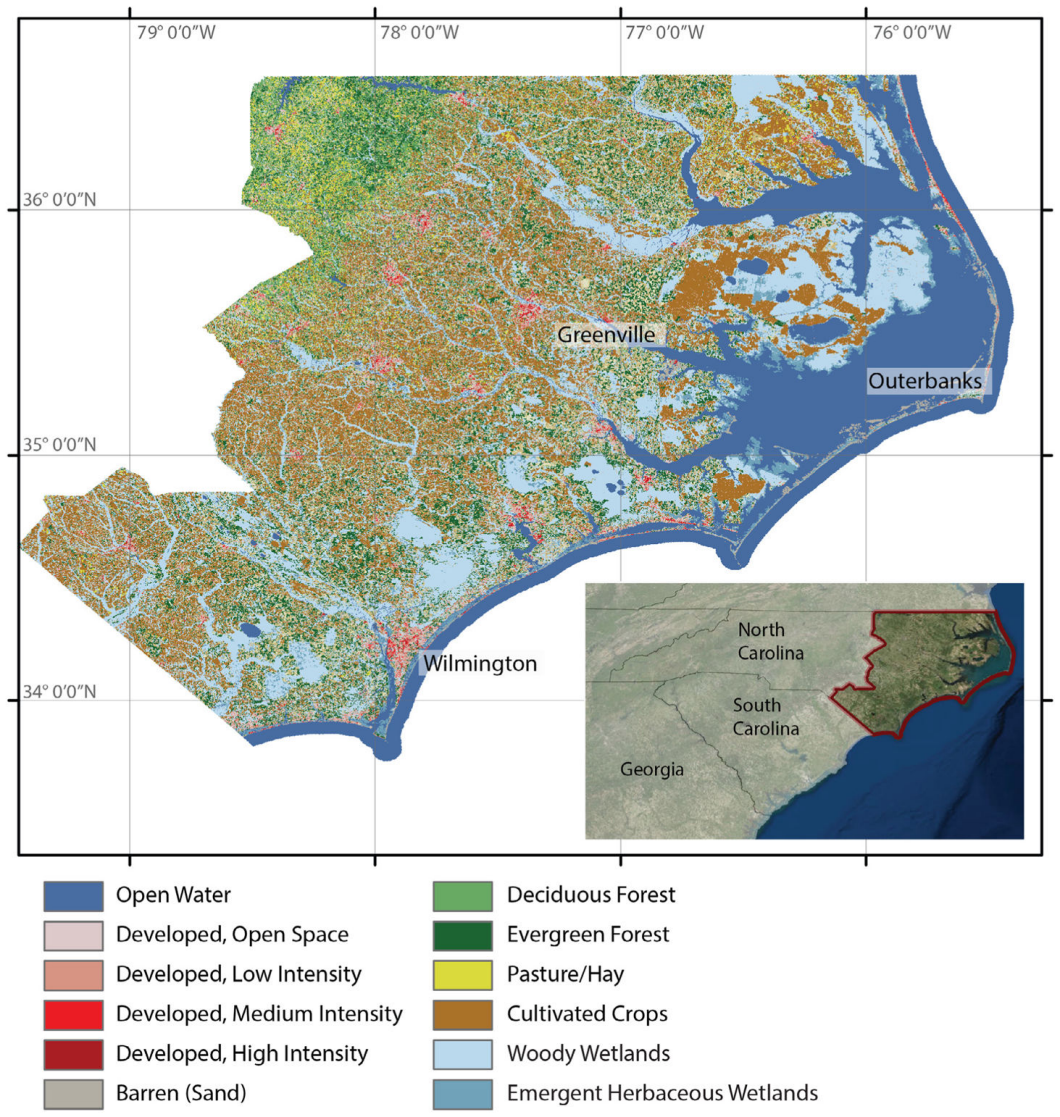
Map of land-use and major urban center of the case study region within North Carolina, US. Sources: Esri, DigitalGlobe, GeoEye, i-cubed, USDA FSA, USGS, AEX, Getmapping, Aerogrid, IGN, IGP, swisstopo, and the GIS User Community

The Middle Atlantic Coastal Plain in North Carolina is a low-lying area comprising agricultural lands and approximately 484 km (301 mi) of coastline (Fig. 1). The area is home to a variety of ecological communities, including tidal and coastal marshes, flood-plains, swamps, peatlands, and barrier islands. The coastal beaches, charismatic species and cultural history contribute substantially to the regional economy through tourism expenditures with an estimated 4.6 billion U.S. dollars spent annually (Association, 2013). These iconic landscapes are increasingly threatened by human population growth, amenity migration to coastal regions and unsustainable land-use practices (Bin et al., 2005). Compounding these stressors are additional factors related to climate change – particularly direct inundation from sea-level rise, saltwater intrusion and coastal forest retreat that are threatening terrestrial and non-terrestrial habitats (Chen et al., 2017; Engelhart et al., 2009; Hauer et al., 2016). Substantial public and private funds have been allocated to conserve tourist attractions that support local economies, for example, through beach nourishment and stabilization programs and the relocation of culturally significant structures (Bin et al., 2005). Identification of locations that are important to the public, and the characteristics associated with these special places, offers the opportunity for targeting projects that would enhance and conserve important cultural ecosystem services.

### 2.2. Data collection and processing

To assess highly visited locations, we collected all photographs uploaded to the Panoramio website for the years 2005–2015 using the website application programming interface (API). We collected all data using a python code that systematically mined the geographic coordinates included with the social media posts on the website (van Zanten et al., 2016a). This ensured that we captured all content uploaded for the case study region and the adjoining 3 km region that could possibly view our study site (n = 16,582). Unlike more popular platforms such as Facebook and Instagram, which have placed limits on content access, Panoramio data are accessible to the public. Panoramio also offers highly accurate locational data compared to alternative social media like Flickr (Zielstra and Hochmair, 2013). We sought this high accuracy to assure that viewsheds reflected the actual visible-sensory environment of photographers. In addition to collecting the geographic coordinates associated with content contributed by the public, we also obtained the Panoramio urls from which photos could be downloaded. Coordinates were then mapped to build a geographic database of the precise locations of photographic capture. We decided not to eliminate multiple photographed locations as done in other social media studies (van Zanten et al., 2016b; Wood et al., 2013) to prevent active user bias, as evaluation of the number of multiple photographs taken by the same individual on the same day was low (mean = 3.9, std. dev. 7.3). We also wanted to capture the various landscapes experienced by individuals on their visits to the region.

We constructed individual viewsheds for each photo location using a digital surface model (DSM) interpolated from LiDAR data (Program, 2014). We developed a 6.096 m by 6.096 m resolution DSM for the entire case study area from the North Carolina Flood-plain Mapping Program LiDAR product (Neteler and Mitasova, 2013). The DSM was created by binning 240 billion LiDAR points. The maximum Z value in each cell was calculated with an upper boundary of 300 m to remove high-noise outliers. With the high-resolution DSM, we identified structures and buildings visible to the individual or group taking the photograph (Vukomanovic and Orr, 2014). Such a resolution improves identification of specific structures and elements present in visitors’ photographs. While a few studies have leveraged viewsheds for assessing CES (Schirpke et al., 2016; Yoshimura and Hiura, 2017), to the authors’ knowledge this is one of the first studies using high resolution LiDAR-derived DSMs (Vukomanovic et al., 2018). Viewshed calculations were based on an assumed human height of 1.6 m (5 ft 3 in). We chose a max view range of 3 km, based on observed visibility for several (n = 10) test sites in the study area determined using Google Earth. We also draped 2011 National Land Cover Data (NLCD) (Homer et al., 2015) over the DSM for analysis of the land cover composition experienced by visitors Table 2 and validation of photo locations (described in Sections 2.4 and 2.3). All mapping and LiDAR calculation were done using GrassGIS (Neteler and Mitasova, 2013). Viewshed calculation were automated using a python scripthttps://github.com/ptabriz/Viewshed_analysis.

### 2.3. Contextualizing & validating visitor experience

To validate the database, we assessed both the accuracy of the locations of the volunteered photographs and their content. Users of Panoramio contribute photos to the website and can volunteer locations (i.e., coordinates) of their photo through an interactive web-portal, which may result in geographic misclassification (Zielstra and Hochmair, 2013). We also wanted to assess if all photos depicted content and activities relevant to the study of CES. To evaluate image content, we manually classified a random subsample (n = 1708; approx. 10%) of all photographs into non-mutually exclusive categories, visually assessing them for the presence of outdoor activities, historical monuments and coastal attractions, plants or animals, sunset/sunrise and weather phenomena, as well as the land use/cover for the purpose of validation. We chose these broad categories instead of the common CES classification schema, which can suffer from assessor’s perception bias due the subjective nature of the definitions (Oteros-Rozas et al., 2017). We assumed that high levels of photography indicated locations offering multiple CES, often in combinations that are difficult to define due to their subjectivity (van Zanten et al., 2016a). This sampling approach was chosen due to the challenge of adequately visually assessing all photographs. It was also determined that a majority of photographs fit within our broad CES categories (86.8%), making filtering of the database unnecessary. To assess geographic accuracy, we compared the land use/cover shown in the images with the land use/cover that was determined to be visible from the viewshed of each point, calculating percentage agreement and Cohen Kappa Coefficient, which accounts for the possibility of the agreement occurring by chance (Cohen, 1968).

### 2.4. CES analysis

We further analyzed an aggregation of all viewsheds to evaluate the landscape characteristics associated with the most photographed locations, which we define as viewshed intensity. We constructed an overlay of the 16,582 viewsheds to map the cumulative viewing area of all of the photographs, where locations of overlap between viewsheds represented the locations that experienced relatively more views. While using all viewsheds resulted in including both relevant and non-relevant photographs, we determined that the small proportion of non-relevant photographs (13.2%) would not impact our mapped results. The viewshed intensity map was then matched with different spatial layers representing landscape features and characteristics that might explain such photographic intensity. Model analysis of the layers was based on a 1% random sample of cells for the entire study region (n = 1,000,000 cells representing 30 km^2^ of the total 2251.7 km^2^). This approach was chosen to balance computational efficiency and geographic representativeness. Measuring landscape appreciation by way of viewshed is unlike previous social media studies evaluating landscape values that have primarily used coarse aggregation, (e.g., 5 km cell) of photo intensity (van Zanten et al., 2016a) and therefore assuming a more uniform environmental experience. Our viewshed method more closely reflects the environment experienced by visitors (Garcia-Palomares et al., 2015) at the landscape scale.

To test landscape factors contributing to intensity of views, we fit a model where the response variable was assumed to follow a negative binomial (NB) distribution using the R statistical package lme4 (Bates et al., 2014). NB is a general form of the Poisson regression model that can account for numerous zeros (i.e., overdispersion) and relaxes the requirement that the variance match the mean in the dependent variable. The negative binomial distribution is given by Scott Long (1997):

(1) $$\frac{\gamma (y+1/\alpha )}{\gamma (y+1)\gamma (1/\alpha )}\;{\left(\frac{1}{1+\alpha \mu }\right)}^{1/\alpha }\;{\left(\frac{\alpha \mu }{1+\alpha \mu }\right)}^{y}$$

where:

(2) $$ln\mu =\beta_{0}+\beta_{1}x_{1}+\ldots \ldots +\beta_{k}x_{k}$$

is the regression function that estimates the cumulative viewshed intensity (i.e., number of overlapping viewsheds) based on *k* independent variables (Scott Long, 1997). The 1/α term models the difference in variance from what would be assumed in a Poisson regression. The alpha (α) was selected by a systematic iterative check of multiple terms and comparison of the Akaike information criterion (AIC), which is an estimator of the relative quality of models given a set of data (Overmars et al., 2007).

We tested the strength of different independent variables for explaining viewshed intensity using a number of spatial layers representing landscape characteristics of both the natural and built environment (Table 1). Landscape and vegetation cover have been shown to influence the scenic qualities of coastal areas (Ergin et al., 2006) and landscapes in general (Van and Verburg, 2014; van Zanten et al., 2016a); therefore we included representative land-cover classes of the region including beaches, forests, agriculture, wetlands, and urban areas (Table 1). Water, an aesthetically pleasing landscape feature and a potential recreation area, is included using a logarithmic transformation of distance (log) to coastlines and the location of fresh and ocean water bodies (Artell, 2014). Log transformation increases the weight of near proximity distance measures to features (e.g., coastline), simulating the assumed nonlinear relationship (i.e., photographs are taken at or near features rather than increasing with proximity). We distinguished between fresh water and the sea to account for the high levels of beach tourism in the region. Locations within protected areas (federal and state parks) and in proximity to hiking trails (100 m buffer) were used to account for the assumed attraction of these areas for visitors. Likewise, we included distance in kilometers (log) to coastal (e.g., beach piers, kayak rentals), and historical attractions (e.g., commemorative monuments). These features represent qualities that might enhance the visitor experience of the landscape (Van Berkel et al., 2014) and contribute to its uniqueness (Hausmann et al., 2016). We also included slope to account for terrain that may represent popular lookouts and vistas. Finally, we used population density to control for the increased number of photos that occur where people live. A test for multicollinearity (i.e., variance inflation factor) indicated that travel time to urban centers and distance to popular fishing locations were correlated with urban lands and distance to lakes respectively and therefore we omitted the latter variables.

Moran’s I tests of spatial autocorrelation did not indicate the presence of a spatial relationship within the residuals of our model estimates (Fig. S2), which can result in incorrect error probabilities that can highly influence coefficient estimates (Anselin and Rey, 1991). We present standardized coefficients to indicate the relative influence effect of the variables within the model. Standardized coefficients assesses how many standard deviations a dependent variable will change, per standard deviation increase in the predictor variable. Goodness of fit was estimated by calculating McFadden and Nagelkerke pseudo *R*^2^. Pseudo *R*^2^ values compare the maximum likelihood of the model with a nested null model fit (Nagelkerke, 1991). McFadden pseudo *R*^2^ estimates between 0.2 and 0.4, and Nagelkerke estimates between 0.7 and 1.0, indicate a model where the variance is well captured (i.e., a well-fit model).

## 3. Results

### 3.1. Overview

**Fig. 2:**
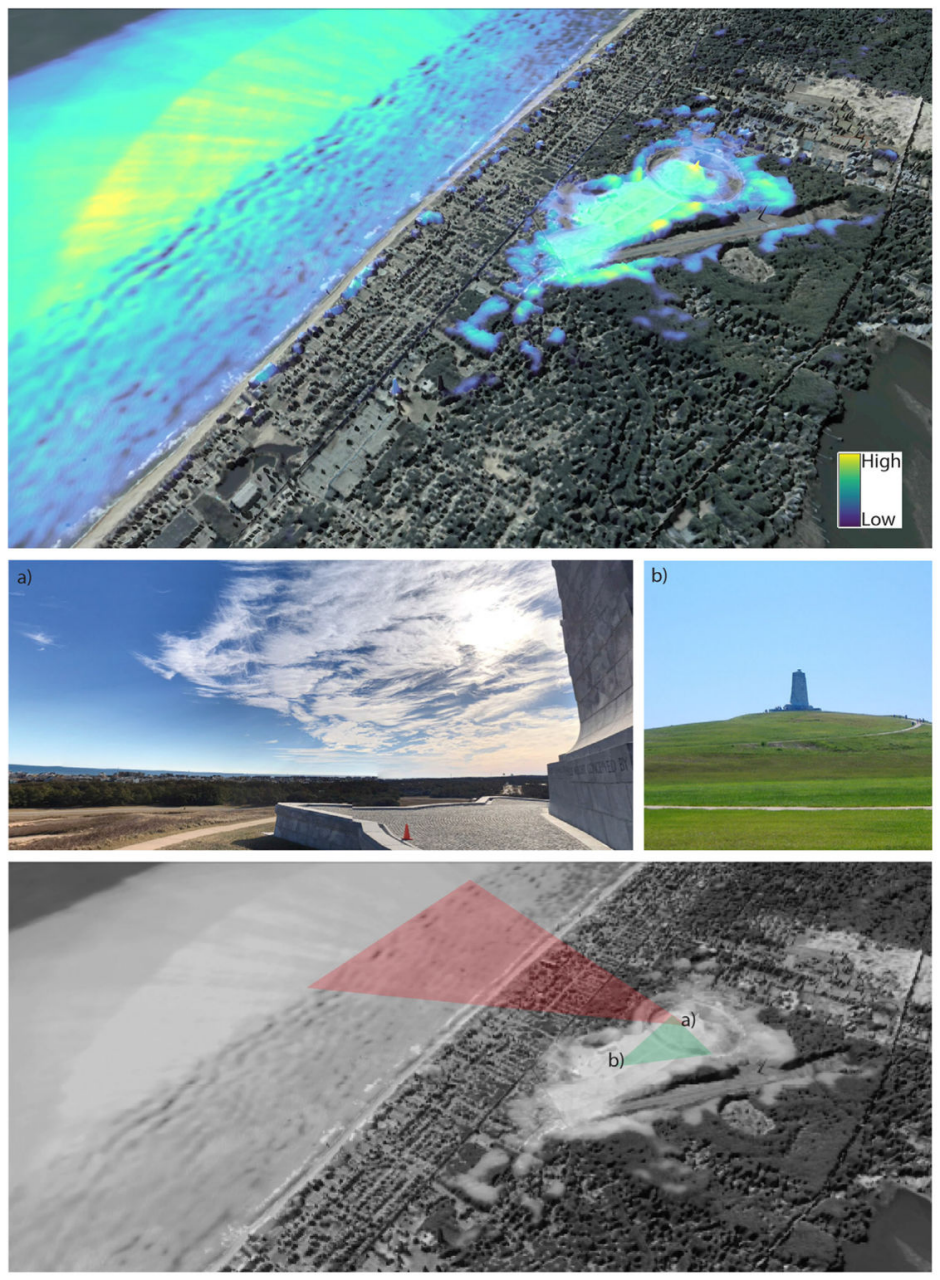
Example of aggregated viewsheds using a 3D rendering of the terrain draped over an aerial photo (top). The Wright Brothers National Memorial is distinguishable as the yellow inland peak, while the ocean also had numerous views. Example photographs contextualized locations where individuals captured images from the elevated monument (a) and from the site grounds (b). The final image conceptualizes the approximate location and visible areas from these photos.

A total of 16,582 unique photographs were uploaded within the study area from 2005–2015 (Supplemental Material Table S1 & Fig. S1). Aggregated viewsheds reveal popular tourist locations, such as the Wright Brothers National Memorial (Fig. 2;https://sketchfab.com/models/75d3cc4c556b4451ac7a460cd3be97b2), and other specific hot spots of photography. Three-dimensional rendering of the entire dataset can be explored online athttps://sketchfab.com/models/69178d2b5c384e8d99d9056e7c5ae2a1.

### 3.2. Viewsheds and photo content

**Fig. 3:**
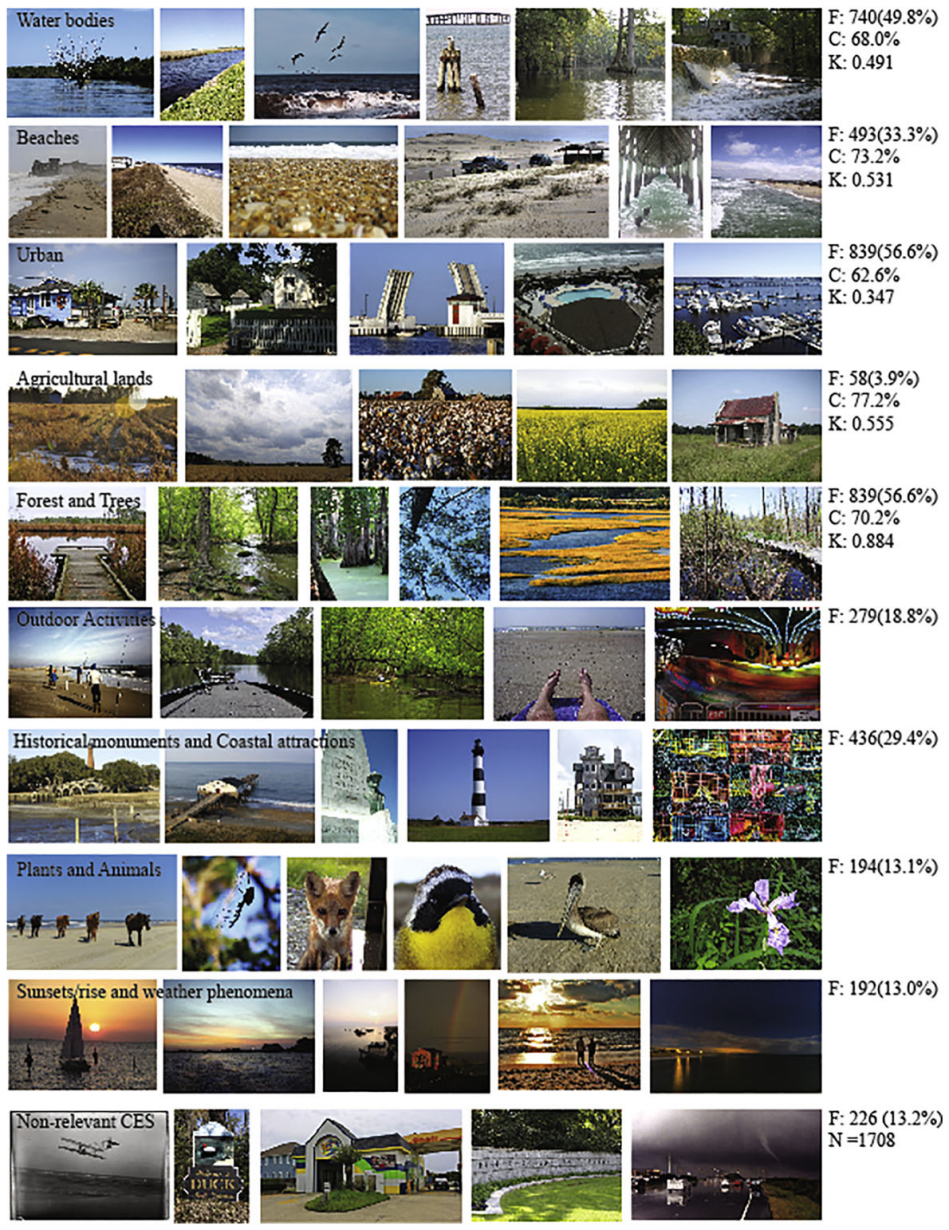
Frequency (F) of photographs (n = 1708) depicting defined land use, and landscape feature classes. Correspondence (C) and Cohen’s Kappa (K) measurements comparing photographic classes of land use with the corresponding viewshed land use for validation.

Our analysis of social media photo-content revealed a diversity of natural and built environments and numerous nature-based activities (Fig. 3). However, we also found that 226 (13.2%) depicted advertisements, scanned historical photos, and photos of businesses and outcomes of natural disasters that likely do not reflect an appreciation of the location indicated. Of the total relevant photographs (1482, 86.8%), 18.8% depicted outdoor recreational and leisure activities, 13% included sunsets/sunrises and weather phenomena, 13.1% contained plants and animals, and 29.4% historical monuments and coastal attractions (Fig. 3).

A large proportion of the photographs included water bodies (49.8%), the beach (33.3%), and urban structures related to beach tourism in the region (56.6%) (Table 2). Analysis of percentage land-cover from the viewsheds similarly indicated that open water, low- to medium-density developed urban areas, and beaches (barren class, which includes sand; Table 2) were frequently visible features from locations contributed by the public through their social media photographs. Woody and emergent herbaceous wetlands and evergreen land cover were found to be relatively visible for all viewsheds (mean 8.0%, 5.1% and 50.2% respectively), unlike our findings from the photograph content analysis. The challenge of distinguishing between wetland forest and forests in photographs likely contributed to misclassifications resulting in underestimating the wetland cover class and overestimating the forests (56.6%). Agricultural lands were not often photographed (3.9%) despite contributing to a share of the total percentage visible area (cultivated crops: 4.1%; pasture hay: 0.6%). The relatively large proportion of cultivated land in the study area likely accounts for this overestimation, where the viewshed calculations indirectly capture this land use in the 360° visibility assessment. Other frequently photographed features including tourist attractions (29.4%), plants and animals (13.1%), sunsets (13.0%), and outdoor activities (16.3%) were not captured in the uniqueness of the location.

Analysis of the correspondence of the locations of the photos with actual photo content indicated moderate to high accuracy (Fig. 3). Correspondence of the land cover visible in individual viewsheds with photo content from these locations ranged from a low of 62.6% for urban areas to a high of 77.2% for agricultural (Fig. 3; C). Estimates of Cohen Kappa (Fig. 3; K), that take into account the possibility of the agreement occurring by chance, ranged from moderate for the presence of urban (0.347) in both photographs and viewsheds to almost perfect for the same measure considering forest (0.884). As our viewshed calculation is based on a 360° visible area that is rarely achievable in photographs, we expected moderate agreement comparing the two datasets. Disagreement for urban areas is likely related to individuals avoiding urban elements in their photographs despite being near or located in the urban setting and the coarse resolution (30 m) of the land cover maps that may not capture all structures. Overall, validation suggests good accuracy of the photo locations, which can provide a relatively reliable empirical source of data on locations of CES and the associated aesthetic appeal, public enjoyment of outdoor activities, and sense of place.

### 3.3. Cultural landscapes

Model estimates of the landscape characteristics associated with viewshed intensity were well fit (Table 3) as indicated by the pseudo *R*^2^ measures (Nagelkerke *R*^2^: 0.72 and McFadden *R*^2^: 0.31). Model findings indicate that the ocean, beaches, national and state parks, and trails were positively associated with the count of viewshed intensity (Table 3). Proximity to coastlines, and coastal or historical attractions were likewise significantly positively associated with these highly viewed and visited locations. For example, being located on the beach increased views 9.9-fold compared to other locations and viewshed intensity increased by 50% for every 1 km proximity (log) to the coast. Being located on a lake or river, trail, or a National or State Park, increased the probability of views by 16.8, 1.3, and, 1.7 respectively. Higher population density and being located in an urban area also increased viewing probability, due to numerous social media posts originating in these areas. Forests, agricultural lands, impervious areas, and wetlands were negatively associated with the number of views. Woody emergent wetland is particularly unpopular as the incident rate was a mere 0.2 compared to all other areas.

## 4. Discussion

In this paper, we presented a methodology for empirically quantifying the visual and sensory landscape qualities that contribute to CES using photograph locations and content contributed by the public via a social media platform. By leveraging high-resolution LiDAR data to construct viewsheds and classification of volunteered images, we were able to 1) better define the visible area of these locations; 2) enrich our understanding of the visual sensory qualities that contribute to appreciated CES; and 3) characterize specific natural and built environments and activities that are enjoyed by public. Quantifying CES has often been based on expert evaluation rather than a wide representative sample of the publics’ perceptions (Van and Verburg, 2014; van Zanten et al., 2016b). Empirically based approaches have primarily used coarse uniform areas (i.e., cells) that are assumed to approximate the experienced environment. Our combination of high-resolution viewshed mapping with social media analysis provides important advances that resolve the challenges of capturing and representing how different individuals experience a landscape.

### 4.1. Overview of findings

Our study demonstrated the diversity of ways that social media can be leveraged as a data source for understanding CES. By using multiple techniques, we were able to distinguish the visual-sensory as well as spatial importance of different landscape elements and features. Model estimates indicated the location-specific qualities that are important to the public including beaches, water bodies, slope of the terrain, and coastal attractions (e.g., monuments, piers). While photographic analysis largely corroborated these model findings, difference in results between forest and agricultural lands were apparent, which is discussed further below. Additionally, the photographs capture the plants and animals, weather phenomena, and outdoor activities for which other sensory stimuli can be inferred. Such data give rich qualitative evidence of the multiple qualities that contribute to experience and appreciation of the landscape (e.g., the smell of rain, the song of birds). This presents a unique opportunity for including temporally and spatially variable sensory elements like the presence of wildlife and weather phenomena in the spatial analysis of CES (Van and Verburg, 2014). Photographic evidence of their importance is clear and their variable nature has scarcely been considered in CES studies.

Our findings correspond to similar studies evaluating scenic qualities of coastal areas and landscapes in general (Oteros-Rozas et al., 2017), and advance our understanding of these diverse, interrelated qualities. The results indicated that beach areas are the most visited and photographed locations along the coastal-inland gradient. Model results, photographic content, and viewshed analysis all confirm this popularity. Similar to our model findings, beach terrain (e.g., dunes and cliffs), skyline vistas, and specific built environments have previously been assessed as valuable beach qualities (Ergin et al., 2011). At the landscape scale, the presence of water, protected areas, trails, and forests are preferred landscape features across empirical studies (van Zanten et al., 2016a; Van and Verburg, 2014). The negative effect of agricultural and forested lands in our model was not consistent with other studies of landscape appreciation (Van and Verburg, 2014). Forest results, although counterintuitive considering the large number of photographs with trees, can be explained by forests often acting as visual barriers in a context of relatively flat relief. This is a limitation of using the aggregated viewsheds to model appreciated landscape qualities for some areas. We would argue that in fact forests are appreciated based on the photographic content. A lack of agricultural appreciation was a surprising result especially considering the numerous studies that demonstrate their scenic and recreational appreciation by the public (Van and Verburg, 2014; van Zanten et al., 2016b). Our findings suggest that within the landscape mosaic of our case study, beaches were more valued than agricultural landscapes. The model and photographic content analysis demonstrated the importance of cultural historical features, as well as charismatic plants and animals. While inferred to be important in foundational CES work (Assessment, 2005), landscape biota were uniquely and empirically revealed as important elements in this research. Study of CES has primarily focused on natural features (Gee and Burkhard, 2010) that are appreciated by society, while our results reveal that built features also enhance landscape experiences.

### 4.2. Methodological considerations and challenges

While our analysis allowed us to contextualize landscapes and CES appreciated by visitors over a broad extent, it may not precisely capture appreciated features or the specific services they provided. Extraneous information was likely introduced by including the entire viewshed and some photographs that are not relevant to CES, potentially biasing our results. While the photograph point locations clearly indicate landscapes used by visitors, viewshed analysis captures the 360° visible area in relation to the photographer, including valued and non-valued features. Photographic framing, intentionally and unintentionally, targets features worthy of capture due to their deemed attractiveness or relevance (Dunkel, 2015) and these valued features are difficult to isolate based on viewshed calculations. We do capture highly important locations via the viewshed intensity measure (Yoshimura and Hiura, 2017), though the near proximity of the photographer is also included. Photographs depicting less CES-relevant material were also impossible to filter out due to the challenge of classifying all images. While inclusion of these less valued areas and non-CES-relevant data likely added noise to our analysis, we expect the large number of relevant locations and photographs resulted in model estimates that are generally reflective of public appreciation. We also argue that viewshed intensity captures the entire environment contributing to the visitor experience, consisting of various built and natural landscape qualities and elements that make these locations unique.

We also note that the range of CES captured by this user group may not have been representative of the full array of CES provided by the landscape (Oteros-Rozas et al., 2017; Sonter et al., 2016). For example, people using the landscape for recreation may have been more likely to take photographs than those using the landscape for spiritual purposes. Future work could couple additional viewshed and photo content analysis with surveys to compare the range of CES captured by each method.

### 4.3. Advancing CES research with social media and novel technologies

Landscape preference surveys have relied primarily on visual assessment of landscape elements and features (Ergin et al., 2006; van Zanten et al., 2016b). For example, numerous studies have assessed the amenities and environmental characteristics of highly attractive areas using surveys of public preference employing photo visualizations (Van and Verburg, 2014) and immersive environments (Dockerty et al., 2005; Brown and Fagerholm, 2015; Tabrizian et al., 2018). Such studies largely remove respondents from the landscapes that they are evaluating in controlled studies, which may introduce unknown response biases. While there are continuing debates about the representativeness of social media (Oteros-Rozas et al., 2017), social media content does represent *in situ* measurements of a large, though demographically uncharacterized, proportion of the population across diverse landscapes. This is rarely achievable with traditional data collection techniques. A continuing challenge will be to maintain and gain access to social media data (i.e., Panoramio is no longer active and been integrated into Google Maps Views, which has prevented mining the data) and ‘keep up’ with popular social media sites that represent the public broadly (Dorning et al., 2017). Such a data source will be highly valuable for bringing a uniquely rich qualitative understanding of CES.

The multi-method approach demonstrated in this study was largely possible due to increased access to novel datasets that are poised to transform research in the field of CES (Dorning et al., 2017), including social media posts, high-resolution LiDAR, and address data of tourism businesses. Leveraging these existing, often underutilized data, can be complementary to traditional survey data collection techniques by offering a large amount of observations for study of actual human-environment interaction that can be efficiently and effectively evaluated. Moreover, new analytical techniques are being developed that will enhance the way such data can be evaluated. For example, photographic image recognition algorithms offer the ability to classify numerous photos and may reduce classification errors by the assessor (Xu et al., 2016). We were unable to filter our photographs due to the challenge of classifying such a large number of images. Future studies can leverage image-recognition technology to help in automating this processes and aid in isolating specific activities and nature-based interactions (e.g., hiking, landscape photography) relevant to CES classification (Richards and Tunçer, 2018).

## 5. Conclusions

In this paper, we presented strong evidence of the visual and landscape characteristics associated with special locations on the coast of North Carolina. Application of our integrative approach for identifying appreciated landscapes and their characteristic features could facilitate targeted management for enhancing and conserving valuable cultural ecosystem services. For example, the findings further confirm the importance of maintaining and conserving historical and coastal attractions in a context of high beach tourism. Additionally, the study demonstrates the diversity of landscapes that are attractive to the public. At the landscape scale, people enjoy forested areas as well as open vistas. The presence of plants and animals in photographs, likewise suggests that leaving room for flora and fauna enhances our landscape experiences. In this context, conservation of both natural and cultural resources, which were highly photographed, may aid in sustaining the economy through attracting tourist revenue.

## Supplementary Material

Supp

## Figures and Tables

**Table 1:** Description of spatial variables used in model estimates and the source of this data.

Variable	Description	Source
Forest lands	Forest lands (NLCD classes 41, 42 & 43) per 30-m pixel	Homer et al. (2015)
Agricultural lands	(NLCD classes 81 & 82) lands per 30-m pixel	Homer et al. (2015)
Fresh water bodies	Water bodies (NLCD class 11) and Euclidean distance to these lakes and rivers 30 km	Homer et al. (2015)
Oceans & Coastline	The ocean (derived from NOAA classification) and Euclidean distance to the coastline (30 m)	Kelso and Patterson (2010)
Wetlands	Woody and emergent herbaceous wetlands (NLCD classes 90 & 95) per 30-m pixel	Homer et al. (2015)
Urban	Urban lands (NLCD class 21, 22, 23 & 24) per 30-m pixel	Homer et al. (2015)
National and State Parks	Location of protected areas	Homer et al. (2015)
Slope of Terrain	Degree of inclination based on a digital elevation model 30-m pixel	Gesch et al. (2002)
Dist. to historical attractions	Log transformed euclidean distance to commemorative landscapes, and monuments	DocSouth (2017)
Dist. Attractions	Log transformed euclidean distance to signature attractions (e.g., lighthouses, local eateries) as listed by the NC Travel website	Travel (2017)
Trails	A 50 m buffer of bicycling and walking paths (e.g., greenways) in North Carolina	Stull et al. (2017)

**Table 2:** Mean percentage and standard deviation (Std. dev.) of land cover within viewsheds (n = 16,582) constructed from publicly posted photos on Panoramio.

Land cover/use	Mean %	Std. dev.5 %
Open Water	40.97	40.96
Developed open	8.98	13.16
Developed low	8.63	13.53
Woody wetlands	7.99	14.93
Developed medium	6.11	12.21
Emergent herbaceous wetland	5.13	11.11
Barren(sand)	5.02	11.86
Evergreen	4.48	9.12
Cultivated crops	4.06	12.56
Shrub	3.2	6.97
Developed high	2.23	6.92
Grassland	1.15	4.15
Deciduous	0.86	4.33
Mixed	0.64	2.26
Pasture hay	0.56	3.51

**Table 3:** Results of the negative binomial model were estimated using R (Team, 2000) and the glm package. Standardized estimates were calculated using the lm.beta function (Behrendt, 2014). The goodness of fit measures (AIC: 855088, Nagelkerke: 0.720, McFadden: 0.312 indicate a well-estimated model given this set of variables).

	Estimate	Std. Estimate	Pr(>—t—)
(Intercept)	1.8674		0.0000
Agricultural lands	−0.5405	−0.0361	0.0000
Emergent herbaceous wetland	−0.5918	−0.0164	0.0000
Lakes & River	−2.8201	−0.0601	0.0000
Forests	−0.8103	−0.0478	0.0000
Ocean	2.2893	0.1295	0.0000
Woody wetland	−1.2210	−0.0812	0.0000
Urban	0.1780	0.0067	0.0002
National and State parks	0.5475	0.0178	0.0000
Trails (100 m buffer)	0.2710	0.0047	0.0002
Distance to coastal attractions(log)	−0.5665	−0.0650	0.0000
Dist. ocean coastline(log)	−0.6819	−0.1989	0.0000
Dist. lakes and river coastline(log)	−0.1047	−0.0200	0.0000
Dist historical attractions(log)	−0.3236	−0.0336	0.0000
Slope of terrain	0.0003	0.0308	0.0000
Population density	0.0021	0.0264	0.0000

## Appendix A. Supplementary data

Supplementary data associated with this article can be found, in the online version, athttps://doi.org/10.1016/j.ecoser.2018.03.022.
