# Supplementary material for: Quantifying the visual-sensory landscape qualities that contribute to cultural ecosystem services using social media and LiDAR
Source: Ecosyst Serv. Author manuscript; Available in PMC 2018 Aug 22. (PMC6104849; doi:10.1016/j.ecoser.2018.03.022)
Supplement: Supp [file NIHMS983232-supplement-Supp.pdf]

7. Supplemental Material

County	Total Panoramio photos (2005-2015)
Beaufort	504
Bertie	94
Bladen	150
Brunswick	200
Camden	112
Carteret	1471
Chowan	90
Columbus	310
Craven	1034
Currituck	521
Dare	5017
Duplin	227
Edgecombe	99
Franklin	67
Gates	112
Greene	24
Halifax	85
Hertford	61
Hyde	1126
Johnston	541
Jones	118
Lenoir	174
Martin	41
Nash	133
New Hanover	1074
Northampton	72
Onslow	620
Pamlico	423
Pasquotank	154
Pender	278
Perquimans	12
Pitt	616
Robeson	177
Sampson	120
Tyrrell	143
Vance	45
Warren	77
Washington	102
Wayne	238
Wilson	120
**Total**	**16582**

Table S1: Panoramio social media posts per county

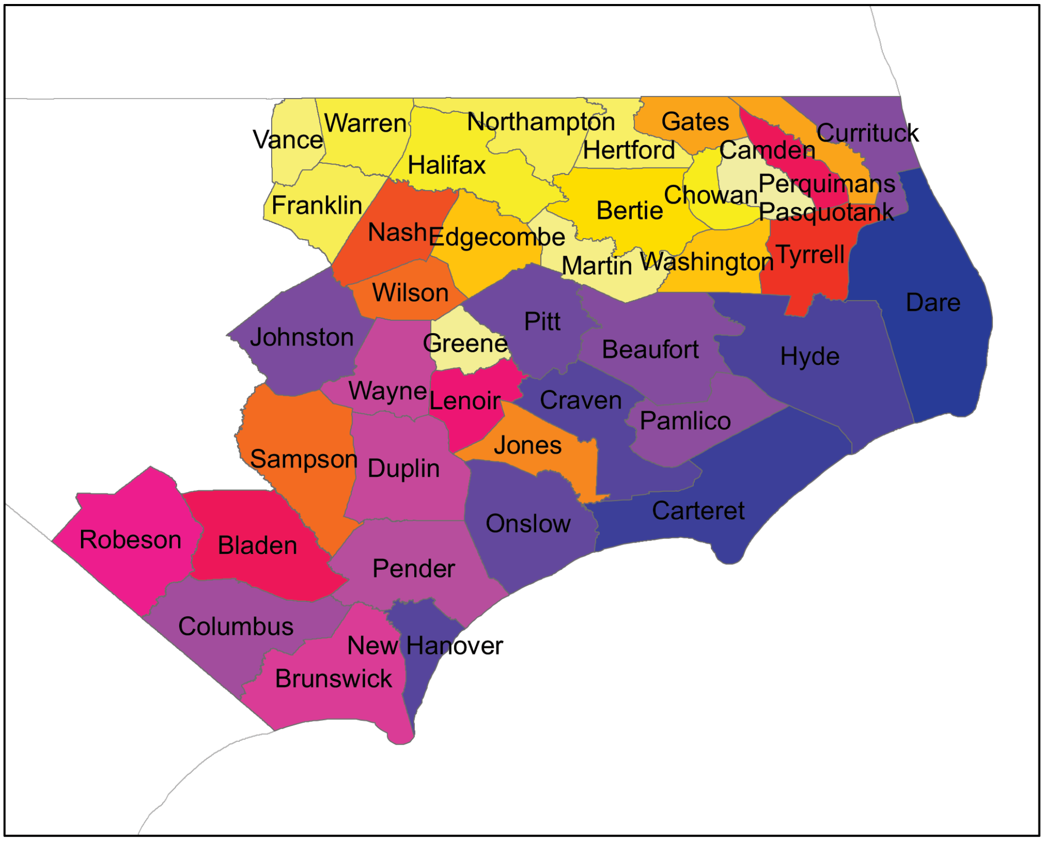

Figure S1: Map of Panoramio social media posts per county

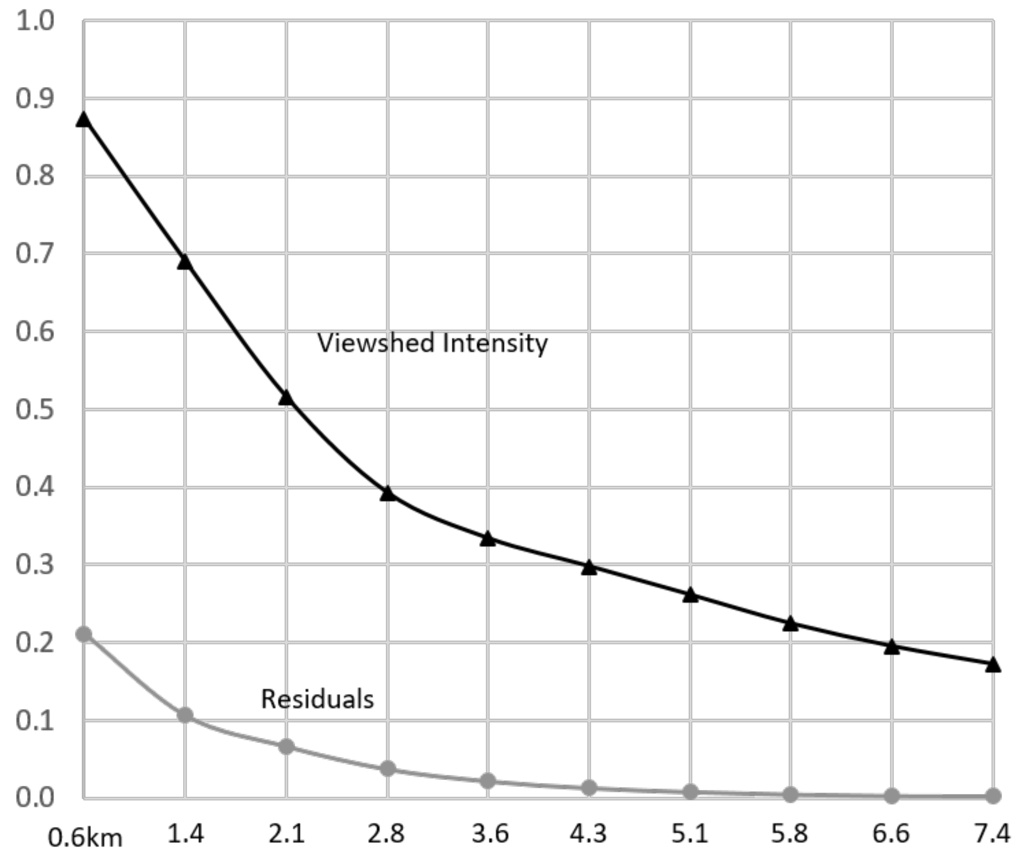

Figure S2: Moran's I estimates for the independent variable cumulative viewshed intensity and the residual of the model estimates. Spatial lag distances are based on the mean euclidean distance from the origin of the random point sample.
